# Measurement of Volatile Fatty Acids in Silage through Odors with Nanomechanical Sensors

**DOI:** 10.3390/bios13020152

**Published:** 2023-01-18

**Authors:** Kosuke Minami, Hisami Kobayashi, Masaaki Matoba, Yuko Kamiya, Subrata Maji, Takahiro Nemoto, Masanori Tohno, Ryoh Nakakubo, Genki Yoshikawa

**Affiliations:** 1 Center for Functional Sensor & Actuator (CFSN), Research Center for Functional Materials, National Institute for Materials Science (NIMS), 1-1 Namiki, Tsukuba 305-0044, Ibaraki, Japan; 2 Institute of Livestock and Grassland Science, National Agriculture and Food Research Organization (NARO), 768 Senbonmatsu, Nasushiobara 329-2793, Tochigi, Japan; 3 Research Center of Genetic Resources, National Agriculture and Food Research Organization (NARO), 2-1-2 Kannondai, Tsukuba 305-8602, Ibaraki, Japan; 4 Institute of Livestock and Grassland Science, National Agriculture and Food Research Organization (NARO), 2 Ikenodai, Tsukuba 305-0901, Ibaraki, Japan; 5 Materials Science and Engineering, Graduate School of Pure and Applied Science, University of Tsukuba, 1-1-1 Tennodai, Tsukuba 305-8571, Ibaraki, Japan

**Keywords:** volatile fatty acids (VFAs), nanomechanical sensors, Membrane-type Surface stress Sensor (MSS), electronic nose (e-nose), silage

## Abstract

The measurement of volatile fatty acids (VFAs) is of great importance in the fields of food and agriculture. There are various methods to measure VFAs, but most methods require specific equipment, making on-site measurements difficult. In this work, we demonstrate the measurements of VFAs in a model sample, silage, through its vapor using an array of nanomechanical sensors—Membrane-type Surface stress Sensors (MSS). Focusing on relatively slow desorption behaviors of VFAs predicted with the sorption kinetics of nanomechanical sensing and the dissociation nature of VFAs, the VFAs can be efficiently measured by using features extracted from the decay curves of the sensing response, resulting in sufficient discrimination of the silage samples. Since the present sensing system does not require expensive, bulky setup and pre-treatment of samples, it has a great potential for practical applications including on-site measurements.

## 1. Introduction

Silage is a type of roughage, which is made of fermented grass or crops to enable long-term storage and a stable supply of feed. The fermentation quality of silage affects not only the feeding system and milk production but also diseases in dairy cows [1]. For example, butyric acid, which is mainly produced by aerobic fermentation, can cause reduced dry matter intake [2], leading to energy metabolism disorder, such as ketosis [3,4]. Therefore, examining the fermentation quality of silage is of great importance to determine whether and how to feed silage. There are two major approaches to examining the quality of silage fermentation: with chemical analysis and with sensory evaluation using human olfaction. Although the sensory evaluation can be performed in the field, its accuracy is limited. Chemical analysis, on the other hand, generally provides high accuracy by quantifying the fermentation products. However, chemical analysis requires expensive instruments and pretreatment procedures, resulting in time consumption and delayed results [5]. In addition, in the case of wrapped silage, the aerobic fermentation proceeds from the moment that the wrap is opened. Therefore, there is a demand for a method to measure the quality of silage fermentation accurately on site.

As sensory evaluations are made on odors of silage, odor is one of the important aspects to determine the quality of silage fermentation. To measure such odors, gas chromatography (GC) is the most common approach, while it also has limitations for on-site measurements. The concept of artificial olfaction—also known as electronic nose (e-nose)—has been proposed using an array of chemical sensors [6] consisting of multiple sensors with different chemical properties. Using this artificial olfaction, a wide range of studies have been reported on the identification, discrimination, and quantification of target odors. Since the size reduction of sensing elements through recent nanotechnology has made the artificial olfaction system small and mobile, potential applications including on-site measurements have been proposed in various fields, such as food, agriculture, environment, medicine, and healthcare [7,8,9,10,11,12,13,14].

In this study, we demonstrate that a nanomechanical sensor array can be utilized for differentiating the silage samples through their odors, especially volatile fatty acids (VFAs), which are one of the key indicators for the silage quality [15]. We used a nanomechanical Membrane-type Surface stress Sensor (MSS) as a platform of artificial olfaction [14,16,17]. By focusing on the relatively slow desorption behaviors of VFAs predicted according to the sorption kinetics of nanomechanical sensing [18,19] and dissociation nature of VFAs, we found that the trends of VFAs in moisture-rich odors can be clearly seen in the decay curve of the signals, regardless of the hydrophilicity or hydrophobicity of the receptor materials. Since the MSS array has various advantages on the on-site measurement [14], MSS-based artificial olfaction provides a promising evaluation platform for the silage quality.

## 2. Materials and Methods

### 2.1. Materials

Polystyrene (PS), polymethyl methacrylate (PMMA), and poly(2,6-diphenyl-1,4-phenylene oxide) (known as Tenax) were purchased from Sigma-Aldrich Inc. and GL Science and used for receptor materials. *N,N*-dimethylformamide (DMF) was purchased from Kanto Chemical Co., Inc. and used as a solvent to prepare solutions of receptor materials for inkjet spotting. Acetic acid (ethanoic acid), propionic acid (propanoic acid), butyric acid (butanoic acid), valeric acid (pentanoic acid), and caproic acid (hexanoic acid) were purchased from Sigma-Aldrich Inc. (Tokyo, Japan), Tokyo Chemical Industry (Tokyo, Japan), FUJIFILM Wako Pure Chemical Corporation (Osaka, Japan), Kanto Chemical Co., Inc. (Tokyo, Japan). All chemicals were used as received. MilliQ water (Merck MilliPore, Tokyo, Japan) was used as water vapor.

### 2.2. Plant Materials and Preparation of Silage

The whole-plant corn (*Zea mays* L.) at the yellow ripe stage, grown in a field at the National Institute of Livestock and Grassland Science (Nasushiobara, Tochigi, Japan), were harvested and chopped into a 32 mm theoretical length size using a forage harvester (Model-790MD; New Holland, New Holland, PA). The plant material was then ensiled in an underground silo. After ensiling, the silage samples with different fermentation quality were chosen and designated as S1-1, S1-2, and S1-3 (the upper part of the silo) and S2-1, S2-2, and S2-3 (the bottom part of the silo), respectively.

### 2.3. Chemical Analysis of Silage Samples

Silage homogenates were prepared as previously described [20] with some modifications. Briefly, 100 mL of distilled water was added to 10 g (fresh weight) of silage material. The resulting solution was homogenized by using a laboratory homogenizer (Pro·media SH-IIM, Elmex). After 5 min of extraction, the sample was filtered through 5A filter paper (Advantec). The resulting eluate was treated with Amberlite (Amberlite IR120H^+^, Tokyo Chemical Industry) and centrifuged at 20,000 × *g* for 5 min. The supernatant was filtered through a membrane filter (pore size 0.45 μm; Advantec) and analyzed with a high-performance liquid chromatograph (HPLC; JASCO Corporation) equipped with Shodex Raspak KC-811 column (8 mm × 300 mm; Showa Denko) and a UV spectrometer (detection wavelength was 450 nm). The column was maintained at 60 °C. The flow rate of the mobile phase (3 mmol L^•1^ of HClO_4_ aq.) was 1.2 mL min^•1^. BTB solution (0.2 mmol L^•1^ of bromothymol blue, 8 mmol L^•1^ of Na_2_HPO_4_, and 2 mmol L^•1^ of NaOH) was used as the reaction mixture.

The water content is 100 less the proportion of dry matter content (%). Dry matter weight of silage was determined with heating material at 60 °C for 48 h. The pH values of the silage extract were determined using a pH meter (Seven Excellence; Mettler-Toledo) attached to an electrode (InLab Expert Pro-ISM; Mettler-Toledo).

### 2.4. Fabrication of MSS

**Figure 1:**
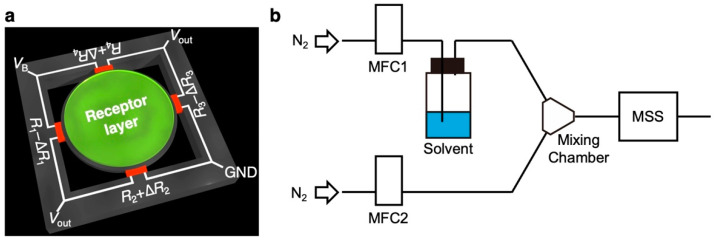
Schematic illustrations of the MSS and its sensing system. (**a**) Configuration of the MSS. (**b**) Schematic illustration of the measurement system.

The construction of the MSS chip and its working principle has been previously reported (Figure 1a) [16,17]. Briefly, MSS consists of a silicon membrane suspended by four bridges, composing a full Wheatstone bridge (Figure 1a). In each bridge, piezoresistors were embedded through boron doping. Receptor materials (i.e., PS, PMMA, and Tenax) were coated on each membrane. When the target gas is introduced to MSS, the receptor layer deforms from sorption of target analytes, such as VFAs, generating surface stress [14]. The surface stress on the membrane is transduced to the four piezoresistive bridges as amplified uniaxial stress, resulting in the changes in the electrical resistance of the piezoresistors embedded in the bridges. The MSS chips used in this study were purchased from NanoWorld AG, Switzerland and provided from Asahi Kasei Co., Ltd. Each receptor material was deposited directly on the membrane of MSS by using inkjet spotter (LaboJet-500SP, MICROJET Corporation, Nagano, Japan) with a nozzle (IJHBS-300, MICROJET Corporation). In the present study, we used commercially available polymers as receptor materials, which exhibit different chemical selectivity. Each receptor material was dissolved in DMF (1 mg mL^•1^), and the solutions were deposited onto each channel of the MSS. A stage of the inkjet spotter was heated at 80 °C during the deposition to promote evaporation of DMF.

### 2.5. Sensing

To estimate the diffusion time constant *τ_s_* of each receptor material to each solvent vapor, we used a sensing measurement system according to our previous work [19] as shown in Figure 1b. The coated MSS chips were placed in a Teflon chamber, which was placed in an incubator with a controlled temperature of 30.00 ± 0.02 °C. The chamber was connected to a gas flow system: a purging line, an injection line, a mixing chamber, two mass flow controllers (MFCs), and a vial for solutions of VFAs (Figure 1b). The vapor of VFAs was produced by bubbling carrier gas. As carrier and purging gases, pure nitrogen gas was used. The duration and the concentrations of the five different VFA vapors were precisely controlled using MFC-1 (injection line) at *P*_a_/*P*_o_ of 0.1, where *P*_a_ and *P*_o_ denote the partial vapor pressure and saturated vapor pressure of the VFAs, respectively. Before measuring MSS signals, pure nitrogen gas was introduced into the chamber for at least 1 min to promote the desorption of molecules adsorbed in the previous measurement. Subsequently, MFC-1 was switched on/off every 10 s with a controlled total flow rate of 100 mL min^•1^ using MFC-2 (purging line) for four injection-purge cycles. Sensing signals of MSS were measured with a bridge voltage of –1.0 V and recorded with a sampling rate of 20 Hz. The data collection program was designed using LabVIEW (NI Corporation).

To measure the silage samples, we used the MSS Standard Measurement module [21] produced by the industry–academia–government collaboration framework called “MSS Alliance” and “MSS Forum” [22,23,24]. The coated MSS chips were placed in a Teflon chamber in the MSS module. The chamber was connected to a gas flow system: a switching valve connected with injection and purging gas lines, a flow meter, and an aspiration pump. The sample and purge gas flows were controlled by the pump with a flow rate adjusted to 30 mL min^•1^. Each silage sample was placed in 20 mL vial and was connected to the injection line. Before measuring MSS signals, pure nitrogen gas was introduced into the MSS module for at least 2 min. Subsequently, the switching valve was switched to the sampling line for 2 min and then switched back to the purging line for 8 min. Sensing signals of MSS were measured at the bridge voltage of –1.0 V and recorded at a sampling rate of 100 Hz.

### 2.6. VFA Profile in Headspace Gas

To measure the VFAs in the headspace gases of silage samples and aqueous solutions of VFAs, PTR-TOF-MS (PTR-TOF 6000 X2, Ionicon Analytik GmbH) equipped with Static Headspace Autosampler was used. The measurement setup was set according to the manufacture’s protocol. The ion source of PTR was operated at a current of 4 mA and a voltage of 145 V with the source-out voltage maintained at 78.56 V. The source valve operating was set at 51%. The voltage, pressure, and temperature of drift tube were maintained at 557 V, 2.8 mbar, and 70 °C, respectively. The *E/N* value, where *E* and N are the applied electric field and the number density of the gas in the drift tube (1 Td = 10^–17^ V cm^2^), respectively [25], was approximately 101 Td. The mass spectrum was recorded in the mass range of *m*/*z* 9–400, and mass calibration was performed using two ion peaks, which are known exact masses, i.e., hydronium ion isotope (H_3_^18^O^+^; *m*/*z* = 21.022) and diiodobenzene fragment (C_6_H_4_IH^+^; *m*/*z* = 203.943). The count rate of primary ion H_3_O^+^, which is calculated from the count rate at *m*/*z* = 21.022 multiplied by 500, was ca. 1.0 × 10^8^ count per second (cps) in this work. The raw mass spectrum obtained from PTR-MS was analyzed using the PTR-MS Viewer ver. 3.3.9.1.

### 2.7. Curve Fitting of the Signal Response

To estimate the diffusion time constant *τ_s_*, we used an analytical solution of nanomechanical sensing based on the sorption kinetics and viscoelastic behaviors as follows [19]:

(1) $$\sigma \left(t\right)=\{\begin{array}{ll} 0, & t<t_{0}\\ -\sigma_{\mathrm{sat}.}+\sigma_{\mathrm{sat}.}\alpha e^{-\frac{t-t_{0}}{\tau_{s}}}\overset{2\left(n-1\right)}{\underset{i=0}{\sum }}{\left(-e^{\frac{T}{\tau_{s}}}\right)}^{i}+\sigma_{\mathrm{sat}.}\left(1-\alpha \right)e^{-\frac{t-t_{0}}{\tau_{r}}}\overset{2\left(n-1\right)}{\underset{i=0}{\sum }}{\left(-e^{-\frac{T}{\tau_{r}}}\right)}^{i}, & t_{0}+2\left(n-1\right)T\leq t<t_{0}+\left(2n-1\right)T\\ \sigma_{\mathrm{sat}.}\alpha e^{-\frac{t-t_{0}}{\tau_{s}}}\overset{2n-1}{\underset{i=0}{\sum }}{\left(-e^{\frac{T}{\tau_{s}}}\right)}^{i}+\sigma_{\mathrm{sat}.}\left(1-\alpha \right)e^{-\frac{t-t_{0}}{\tau_{r}}}\overset{2n-1}{\underset{i=0}{\sum }}{\left(-e^{-\frac{T}{\tau_{r}}}\right)}^{i}, & t_{0}+\left(2n-1\right)T\leq t<t_{0}+2nT \end{array},$$

where *σ*_sat._ and α denote the amplitude of the signal and fitting parameter (see Ref. [19] for more detail); *τ_r_* is the time constant of the stress relaxation for viscoelastic behaviors of receptor materials; *T* and *n* are the duration and the number of injection/purge cycles. The analytical solution derived for multiple injection/purge cycles (Equation (1)) allows us to extract fitting parameters of the current measurements more accurately than previous single injection–purge model [18,19]. To extract values of receptor material properties and diffusion time constant of gases from the experimental data, we used least squares methods with trust region reflective algorithm using Python 3 with SciPy module according to our previous work [19]; *σ*_sat._, *τ_s_*, *τ_r_*, *E_U_*/*E_R_*, and *t*_0_ were extracted using Equation (1) (see Ref. [19] for more detail).

### 2.8. Pattern Recognition Analysis

**Figure 2:**
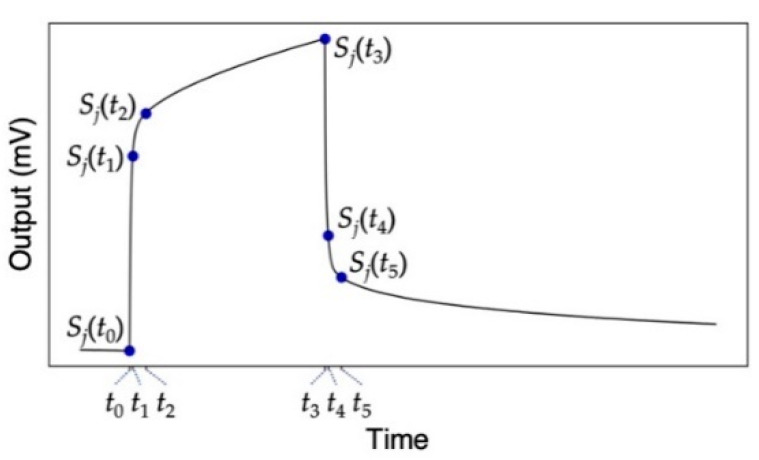
A schematic diagram of the five parameters extracted from each response curve.

To discriminate the silage samples, principal component analysis (PCA) was used for reducing dimensionality of the dataset. By projecting the data onto a lower-dimensional space, one can visually recognize each silage sample according to the cluster separation. The following parameters *S_ij_* were extracted from each MSS response as features for PCA:

*S_ij_* = *S_j_*(*t_i_*) – *S_j_*(*t*_0_) with *i* = 1, 2, 3, 4, 5 (2)

where *S_j_*(*t*) and *t*_0_ denote a signal output of the *j*th receptor material at time *t* and the time when the signal response starts to rise (Figure 2). In this study, we chose four time points for *t_i_*; *t*_1_ = *t*_0_ + 2 [s], *t*_2_ = *t*_0_ + 10 [s], *t*_3_ = *t*_0_ + 120 [s], *t*_4_ = *t*_3_ + 2 [s], and *t*_5_ = *t*_3_ + 10 [s]. Three sets of the five parameters were extracted. PCA was adopted using scikit-learn packages for Python.

## 3. Results

### 3.1. Silage Samples Preparation and Their Headspace Gas Concentrations

During silage fermentation, several compounds are produced such as VFAs and non-volatile lactic acid [1,2]. VFAs, such as acetic acid and butyric acid, are one of the key indicators to determine the fermentation quality of silage [2]; in general, anaerobic lactic acid fermentation results in good fermentation quality, whereas aerobic butyric acid fermentation (alteration) results in poor fermentation quality. To demonstrate the identification of silage with different fermentation qualities with an MSS array, we prepared corn silage in a silo and collected three silage samples from two different positions: the upper and the bottom. The results of the chemical analysis are listed in Table S1. Various VFAs including propionic acid were measured, while valeric acid was not detected with chemical analysis. The concentrations of VFAs in the headspace gas of each silage sample were measured with PTR-TOF-MS (Table 1). The headspace gases of silage samples used in the present study contained not only acetic acid, propionic acid, and butyric acid but also trace amount of valeric acid. Comparing the two different silage samples, the silage collected from the bottom (S2) yields higher VFAs than the silage collected from the upper (S1). Specifically, for the major VFAs in silage such as acetic acid and butyric acid, the concentrations in S2 were approximately 4.2 and 1.7 times higher than those in S1. These silage samples, in addition to water vapor, were used for the discrimination of silage samples through their odors using an MSS array.

### 3.2. MSS Responses to Headspace Gases of Silage Samples

**Figure 3:**
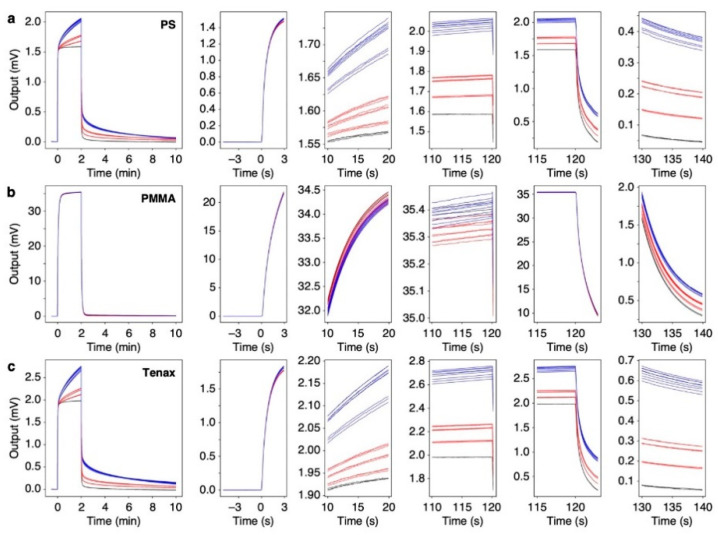
Signal responses to silage samples. Each panel from the top to the bottom shows the signal response measured with MSS coated with PS (**a**), PMMA (**b**), and Tenax (**c**), respectively. Magnified signal responses around *t*_0_ = 0 [s], *t*_1_ = 2 [s], *t*_2_ = 10 [s], *t*_3_ = 120 [s], *t*_4_ = 122 [s], and *t*_5_ = 130 [s] are shown on the right. Red, blue, and black lines correspond to silage 1 (S1), silage 2 (S2), and water, respectively.

To investigate the response patterns to the VFA-rich silage samples, we prepared an MSS array coated with three different receptor materials: PS (hydrophobic material), PMMA (hydrophilic material, sensitive to water), and Tenax (hydrophobic material, widely used as a trapping material) [14,19,21]. Using the MSS array, two sets of silage samples were measured. Each silage sample was measured three times. All signal responses are shown in Figure 2 (see also Figure S1). As can be seen from Figure 2 and Figure S1, signal responses obtained from each sample are highly reproducible. The MSS coated with the hydrophilic receptor, i.e., PMMA, results in similar responses for all silage samples because of the dominant influences of water vapor on the sensing signals, since the major component in silage headspace gases is water vapor. In contrast, the MSS coated with the hydrophobic materials, i.e., PS and Tenax, yielded different signal patterns for each silage sample owing to its low affinity to water vapor. In the cases of PS- and Tenax-coated MSS (Figure 3a,c), the rising curves just after the sample gas injection at 0 s showed similar trends for all samples, while significant differences were observed for each sample from about 10 s after the injection. Unlike the hydrophobic receptors, PMMA-coated MSS showed no significant differences in the rising curve (Figure 3b). In contrast to the rising curves, significant differences are observed in the decay curves not only for the hydrophobic receptors but also for the hydrophilic receptor (Figure 3).

**Figure 4:**
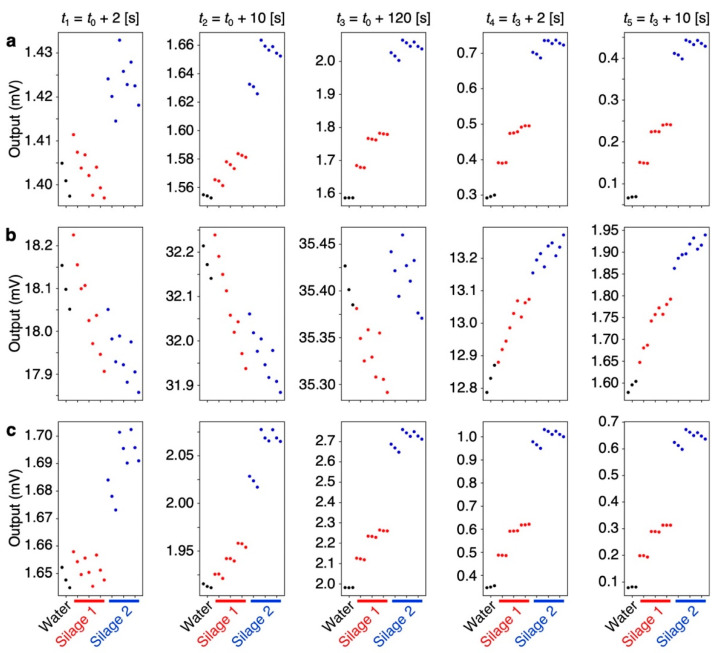
Features extracted from each signal response. Each panel from the top to the bottom shows the plots of output extracted from the signal responses of PS (**a**), PMMA (**b**), and Tenax (**c**), respectively. Each panel from the left to the right shows the output at *t*_1_ = *t*_0_ + 2 [s], *t*_2_ = *t*_0_ + 10 [s], *t*_3_ = *t*_0_ + 120 [s], *t*_4_ = *t*_3_ + 2 [s], and *t*_5_ = *t*_3_ + 10 [s], respectively.

**Figure 5:**
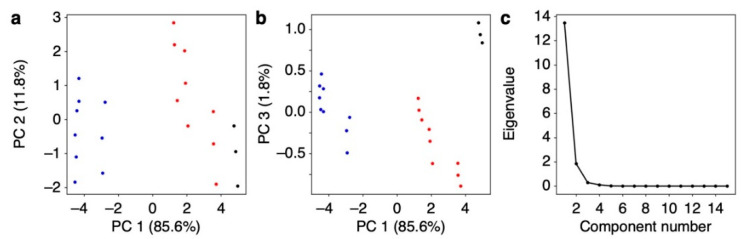
Principal component analysis (PCA). (**a**,**b**) PCA score plots of two sets of silage samples listed in Table 1 with principal component (PC) 1–2 (**a**) and PC 1–3 (**b**). Red, blue, and black correspond to silage 1 (S1), silage 2 (S2), and water, respectively. (**c**), Scree plot of eigenvalues.

From each response curve shown in Figure 3, five output values at *t*_1_ to *t*_5_ were extracted as the features of the sensing signals (Figure 2). The extracted features are summarized in Tables S2–S4. As can be seen in Figure 4, the values extracted at *t*_2_ to *t*_5_ for PS and Tenax and the values extracted at *t*_4_ and *t*_5_ for PMMA yield clear differences for each sample. Although other values are rather scattered, the relative deviations of all signal outputs obtained from each sample compared to the signal intensity (i.e., output value at *t* = *t*_3_) were less than 1% as summarized in Tables S2–S4, indicating that each receptor shows high stability and reproducibility. Using the feature set in Figure 4, PCA was conducted as shown in Figure 5. Scree plots are shown in Figure 4c. Each silage sample and the water vapor are clearly distinguished with well-separated clusters in the principal component space (Figure 5a,b). It should be noted that PC 2 does not contribute to identifying the silage samples. This may reflect a baseline drift of the sensing responses with time, as features with small differences, such as the rising curve of PMMA, contribute more significantly because of the standardization of the feature set.

### 3.3. MSS Responses to Each VFA Vapor

**Figure 6:**
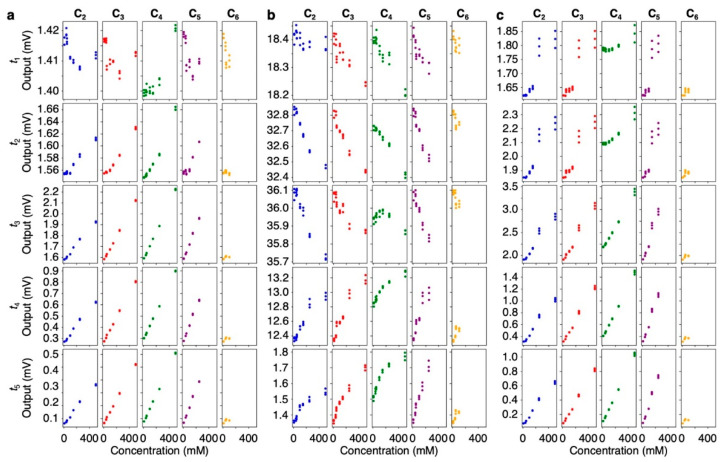
Signal output at *t_i_* of each receptor material to VFAs as a function of VFA concentration in water. (**a**) PS; (**b**) PMMA; (**c**) Tenax. Acetic acid (blue), propionic acid (red), butyric acid (green), valeric acid (purple), and caproic acid (orange) are shown. Each panel from the top to the bottom shows the outputs at *t*_1_ = *t*_0_ + 2 [s], *t*_2_ = *t*_0_ + 10 [s], *t*_3_ = *t*_0_ + 120 [s], *t*_4_ = *t*_3_ + 2 [s], and *t*_5_ = *t*_3_ + 10 [s]**,** respectively.

To investigate the detailed mechanism of the responses of each receptor material to VFAs, we measured MSS responses to the vapors of the aqueous solutions of VFAs. Figures S2–S4 show the signal responses to the series of VFAs with different concentrations in water, which cover the vapor concentrations of VFAs in silage samples (see also Figure S5 for the vapor concentrations measured with PTR-TOF-MS). From the signal responses to aqueous VFAs, the corresponding signal outputs at different time points *t_i_* (*i* = 1–5) were plotted as a function of the VFA concentrations in water (Figure 6). On the one hand, outputs at *t*_2_ to *t*_5_ of hydrophobic PS increase monotonically with increasing concentrations of VFAs, while the outputs at the beginning of the rising curves (i.e., *t* = *t*_1_) show a decreasing trend with respect to the concentrations of VFAs (Figure 5a). On the other hand, most of the outputs during rising curves of hydrophilic PMMA (i.e., *t* = *t*_1_, *t*_2_, and *t*_3_) decrease with increasing VFA concentrations (Figure 6b). This trend probably reflects the decrease of water vapor according to Raoult’s law—the decrease in the mole fraction of water associated with the increase in the concentration of VFAs in water. Compared to the rising curves, the outputs of all receptor materials in the decay curves at *t* = *t*_4_ and *t*_5_ exhibit a linear correlation with the VFA concentrations.

One of the important aspects for explaining such complicated responses is the absorption/desorption behaviors of the target gases. In nanomechanical sensing, an analytical solution was derived based on the first-order sorption kinetics with viscoelastic behaviors of receptor materials in the form of Equation (1) [18,19]. According to the models, one of the parameters (i.e., diffusion time constant *τ_s_*) reflects the absorption/desorption behaviors of the target analyte in nanomechanical sensing [18,19,26]. Considering the first-order sorption kinetics of the single injection–purge model (*n* = 1), the concentration of the target analyte in a receptor layer *C*(*t*) is given with [19]

(3) $$C\left(t\right)=\{\begin{array}{ll} 0\;, & t<t_{0}\\ K_{p}C_{g}\left(1-e^{-\frac{t-t_{0}}{\tau_{s}}}\right), & t_{0}\leq t<t_{3}\\ K_{p}C_{g}\left(1-e^{-\frac{T}{\tau_{s}}}\right)e^{-\frac{t-t_{3}}{\tau_{s}}}, & t_{3}\leq t \end{array},$$

where *K_p_* and *C_g_* are the partition coefficient and gas concentration of the target analyte. When the diffusion time constant *τ_s_* is relatively small and/or the duration *T* is long enough, $e^{-T/\tau_{s}}$ in Equation (3) becomes approximately 0, resulting in the symmetric response between the rising and decay curves.

**Figure 7:**
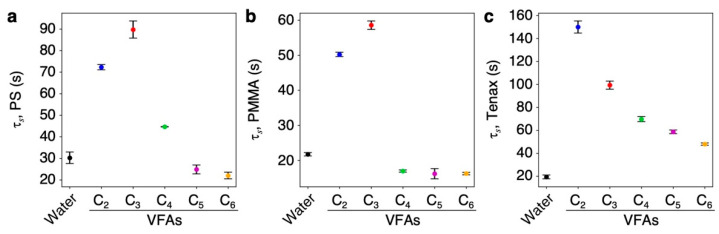
Estimated diffusion time constants *τ_s_* of VFAs to each receptor material: PS (**a**); PMMA (**b**); Tenax (**c**).

To estimate the diffusion time constants *τ_s_* of VFAs and water, we measured the pure vapors of VFAs (i.e., acetic acid, propionic acid, butyric acid, valeric acid, and caproic acid) as well as water. Figure S6 shows the signal responses to the pure vapors of VFAs diluted with pure nitrogen without water vapor. The signal responses to water vapor are also presented in Figure S6 for comparison. Using these signal responses, we apply the fitting of Equation (1) to each response and obtain the diffusion time constant *τ_s_* of the series of VFAs as shown in Figure 7. Some of the diffusion time constants *τ_s_* of VFAs are larger than that of water, indicating the slow desorption of VFAs. In such VFA vapors (e.g., acetic acid), asymmetric behavior is observed between the rising and decay curves, which can be explained by Equation (3), resulting that each cycle of the injection and purge gradually increases (Figure S6). It indicates that a part of the absorbed VFAs remains in the receptor layer during the purge process because the slow desorption requires more time than the purging period (10 s).

## 4. Discussion

In the present study, we demonstrated the discrimination of different silage samples through their odors (i.e., headspace gases) measured with a nanomechanical sensor array. We focused on VFAs, such as acetic acid and butyric acid, produced during silage fermentation because these VFAs are one of the key indicators to assess the fermentation quality of silage [2]. In the silage samples used in the present study, the series of VFAs, including propionic acid and valeric acid, were detected in their odors with PTR-TOF-MS (Table 1), although valeric acid was not detected through chemical analysis (Table S1). The concentrations of VFAs in the odors of one silage group were significantly different from those in the other group of silage, while the concentrations of VFAs in the same group were at similar levels. When these silage samples including water vapor were measured with an MSS array coated with hydrophobic PS, hydrophilic PMMA, and popular trapping material Tenax, the signal responses showed different trends (Figure 3 and Figure 4). By extracting five features from the responses of the MSS coated with each receptor material, the silage samples and water were clearly discriminated with PCA (Figure 5).

In the field of artificial olfaction including MSS-based systems, various feature extraction methods have been proposed [27,28]. We have also reported that it is possible to identify and quantify various analytes using features extracted from their outputs within a couple of seconds of the rising and decay curves [28,29,30,31]. In the present silage samples, however, the features extracted from the beginning of the rising curves (e.g., at 2 s after sample injection, *t*_1_) cannot be effectively used for the discrimination of samples probably because of less correlation with their concentrations of VFAs (Figure 4). In contrast to the rising curves, the features extracted from the decay curves (i.e., *t*_4_ and *t*_5_) clearly show the significant differences reflecting their concentrations of VFAs in silage samples (Figure 4). While the hydrophobic receptors (i.e., PS and Tenax) yield clear differences at *t*_2_–*t*_5_ (Figure 4a,c), the hydrophilic PMMA does not show the significant differences during the sampling process (i.e., rising curve at *t*_1_–*t*_3_) (Figure 4b). Since the major component of the silage headspace gases is water vapor, it is suggested that the rising curves of PMMA are strongly influenced by the water vapor concentrations rather than the differences in the concentrations of VFAs in silage vapors.

To evaluate the trends of silage odors in the signal responses, we also measured the signal responses of aqueous solutions of VFAs varying their concentrations. It is noteworthy that the output of PS at *t*_1_ and the outputs of PMMA at *t*_1_–*t*_3_ tend to be negatively correlated with their concentrations (Figure 6a,b), probably reflecting the decrease in water vapor with increasing VFAs in water, according to Raoult’s law. This trend was also observed in the signal responses of silage samples although it was not a linear correlation with the concentrations of VFAs because mole fraction of water in the silage samples can be varied by not only VFAs but also non-volatile compounds such as non-volatile lactic acid. In contrast to the sample injection period (i.e., rising curve), the outputs at *t*_4_ and *t*_5_ showed strong correlation with each VFA concentration (Figure 6). The reason for the different output trends in the rising and decay curves in Figure 6 can be attributed partially to the slow desorption of VFAs. According to the estimated diffusion time constant *τ_s_* (Figure 7), some of the VFAs obtained relatively large time constants than that of water, resulting in the asymmetric responses of slow desorption VFAs.

**Figure 8:**
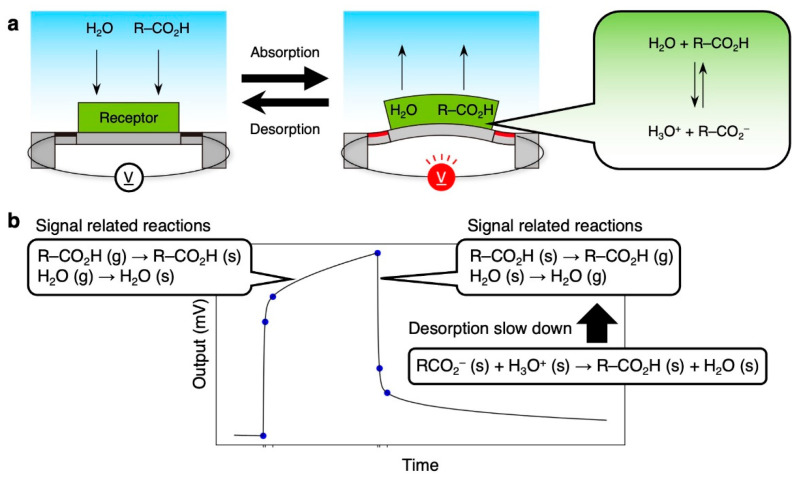
Plausible mechanism of VFA detection. (**a**) Working principle of MSS and their related reactions. (**b**) Signal-related reactions in the absorption and desorption processes.

In contrast to the above-mentioned VFAs, other VFA vapors obtained relatively small *τ_s_* (Figure 7), resulting in the symmetric responses (Figure S6). However, the responses to the aqueous solutions of these VFAs also exhibit asymmetric behavior (Figure 6). Since the asymmetric responses occur only in the presence of water vapor, VFAs in the receptor layer would dissociate into ionic forms in the presence of water. The sensing responses to VFAs in the presence of water can include the following reactions:

(4) $$H_{2}O\;\left(g\right)⇄H_{2}O\;\left(s\right),$$

(5) $$R–{\mathrm{CO}}_{2}H\;\left(g\right)⇄R–{\mathrm{CO}}_{2}H\;\left(s\right),$$

(6) $$R–{\mathrm{CO}}_{2}H\;\left(s\right)+H_{2}O\;\left(s\right)⇄R–{\mathrm{CO}}_{2}^{-}\;\left(s\right)+H_{3}O^{+}\;\left(s\right),$$

where g and s in parenthesis denote the molecule in the gas phase and in the solid phase (i.e., in the receptor layer), respectively. The potential scheme is as follows (Figure 8): (i) during injection process, the sorption of water and VFAs represented by Equations (4) and (5) contributes to the deformation of a receptor layer, while the dissociation process in Equation (6) does not affect the deformation (i.e., does not affect the signal response); (ii) during the purge process, the concentrations of VFAs and water in the receptor layer decrease because of the dissociation process in Equation (6), resulting in the slow desorption of water and VFAs, i.e., a delay in the desorption.

In conclusion, we have demonstrated that an MSS array can discriminate silage samples through their odors in terms of VFAs as indicators. Focusing on the relatively slow desorption behaviors and dissociation nature of VFAs, the resultant large diffusion time constants can be effectively utilized for the discrimination of silage samples. In other words, we have shown that the sensing signal outputs of MSS in the relatively late period in the purge process can be utilized for detecting, identifying, and monitoring various targets in complex mixtures of odors, such as VFAs in silage. This approach should be applicable to other targets such as agricultural and biological aqueous samples because such samples tend to contain gas species with relatively slow desorption behavior. Further, since nanomechanical sensors including MSS can utilize almost any material as a receptor layer, one can select/design/synthesize receptor materials that exhibit distinct features in a certain period during adsorption and/or desorption.

Although the precise determination of the fermentation quality of silage requires not only VFAs but also non-volatile lactic acid, total nitrogen, volatile basic nitrogen, and water content [2], the amounts of VFAs in silage can provide additional information on feed design to maintain the conditions of dairy cows; the amount of butyric acid is related to the probability of ketosis occurrence as well as the activity of Clostridium. Moreover, using precisely selected receptor materials, it has a possibility to measure the volatile basic nitrogen through their odors. The decrease of moisture according to Raoult’s law can also be detected by using hydrophilic receptors, as can be seen in Figure 4b. Therefore, the artificial olfaction system may allow us to evaluate non-volatile components such as lactic acid indirectly by measuring water contents. Machine learning may also support such approaches by highlighting subtle features that may not necessarily appear at specific time points. It should be emphasized that the artificial olfaction system does not require expensive instruments and pre-treatment, such as water extraction, which are used for the conventional chemical analysis, and hence, it has a great potential for on-site evaluation of silage fermentation quality.

## 5. Patents

K.M., H.K., T.N., M.T., R.N., and G.Y. are inventors on WIPO patent application number WO2021/200262, submitted by National Agriculture and Food Research Organization (NARO) and National Institute for Materials Science (NIMS).

## Figures and Tables

**Table 1:** Organic acids in silage samples examined with chemical analysis and their vapor concentrations measured with PTR-TOF-MS. Vapor concentrations are shown in the unit of parts per million (ppm).

Silage	Plant	Acetic Acid	Propionic Acid	Butyric Acid	Valeric Acid
S1-1	Corn (Upper) *^a^*	15.5 ± 4.4	3.72 ± 0.42	9.84 ± 1.11	0.64 ± 0.05
S1-2		31.7 ± 3.2	5.97 ± 0.41	8.78 ± 0.69	0.64 ± 0.01
S1-3		26.1 ± 2.0	5.75 ± 0.42	9.27 ± 0.53	0.74 ± 0.05
S2-1	Corn (Bottom) *^a^*	108 ± 5	8.89 ± 0.60	16.8 ± 0.9	2.09 ± 0.12
S2-2		103 ± 11	7.21 ± 0.66	15.2 ± 1.2	1.56 ± 0.14
S2-3		98.8 ± 7.6	7.27 ± 0.49	15.4 ± 1.2	1.66 ± 0.11

*^a^* Sampling position of silo.

## Data Availability

Not applicable.

## Supplementary Materials

The following supporting information can be downloaded at: https://www.mdpi.com/article/10.3390/bios13020152/s1, Figure S1: Raw signal responses; Figures S2–S4: Signal responses of aqueous solutions of VFAs to each receptor material; Figure S5: Vapor concentration of VFAs in water; Figure S6: Signal responses of pure VFAs; Table S1: Results of chemical analysis of silage samples; Tables S2–S4, Summary of the extracted features from the responses to silage samples.
