# Supplementary material for: Measurement of Volatile Fatty Acids in Silage through Odors with Nanomechanical Sensors
Source: Biosensors (Basel). 2023 Jan 18;13(2):152. doi: 10.3390/bios13020152 (PMC9953262; doi:10.3390/bios13020152)
Supplement: Supplementary file 1 [file biosensors-13-00152-s001.zip › biosensors-2133616-supplementary.pdf]

Supplementary Figures

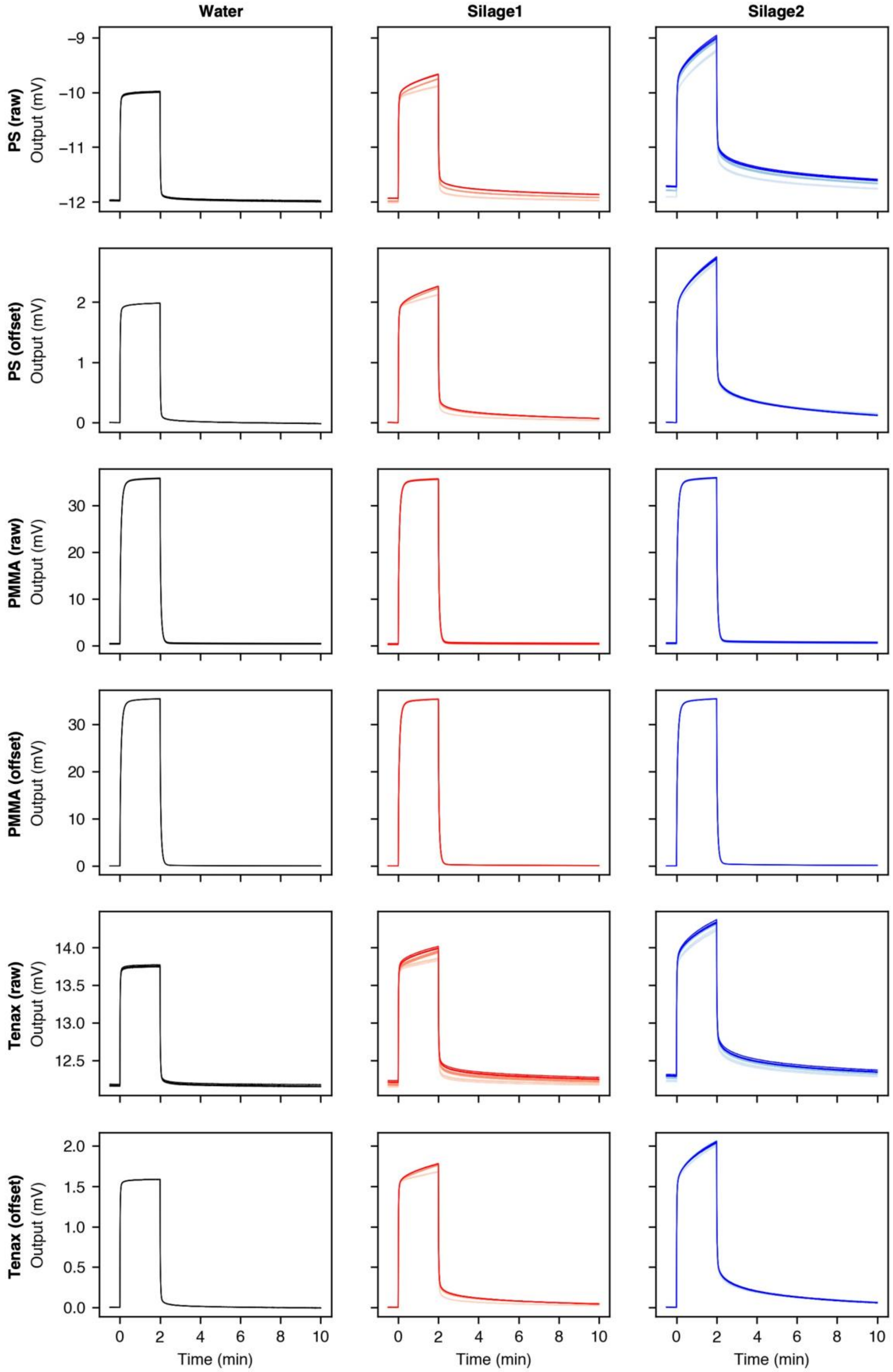

Figure S1. Raw signal responses and offset signal responses to silage samples.

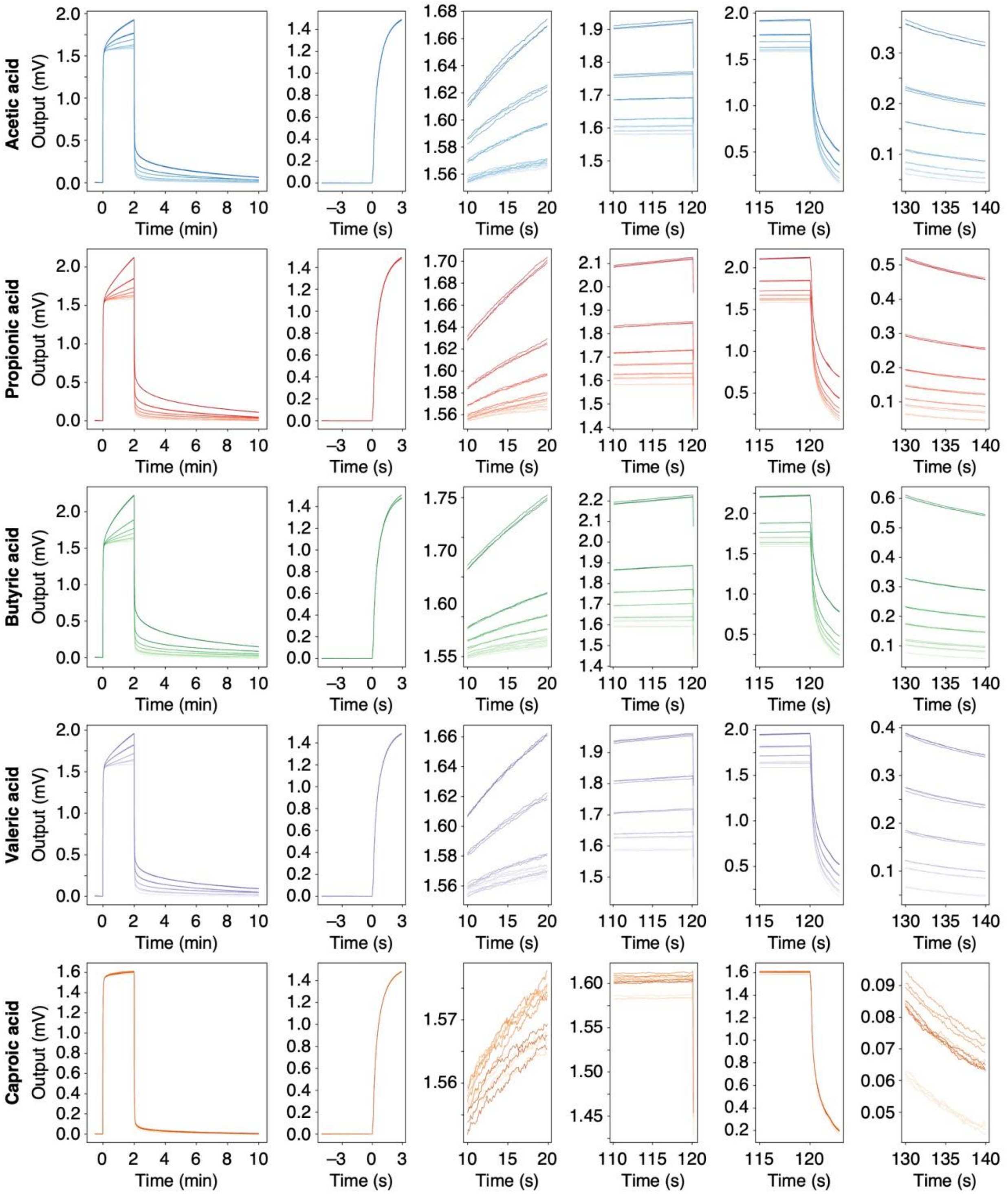

Figure S2. Signal responses of PS to various VFAs with different concentrations in water.

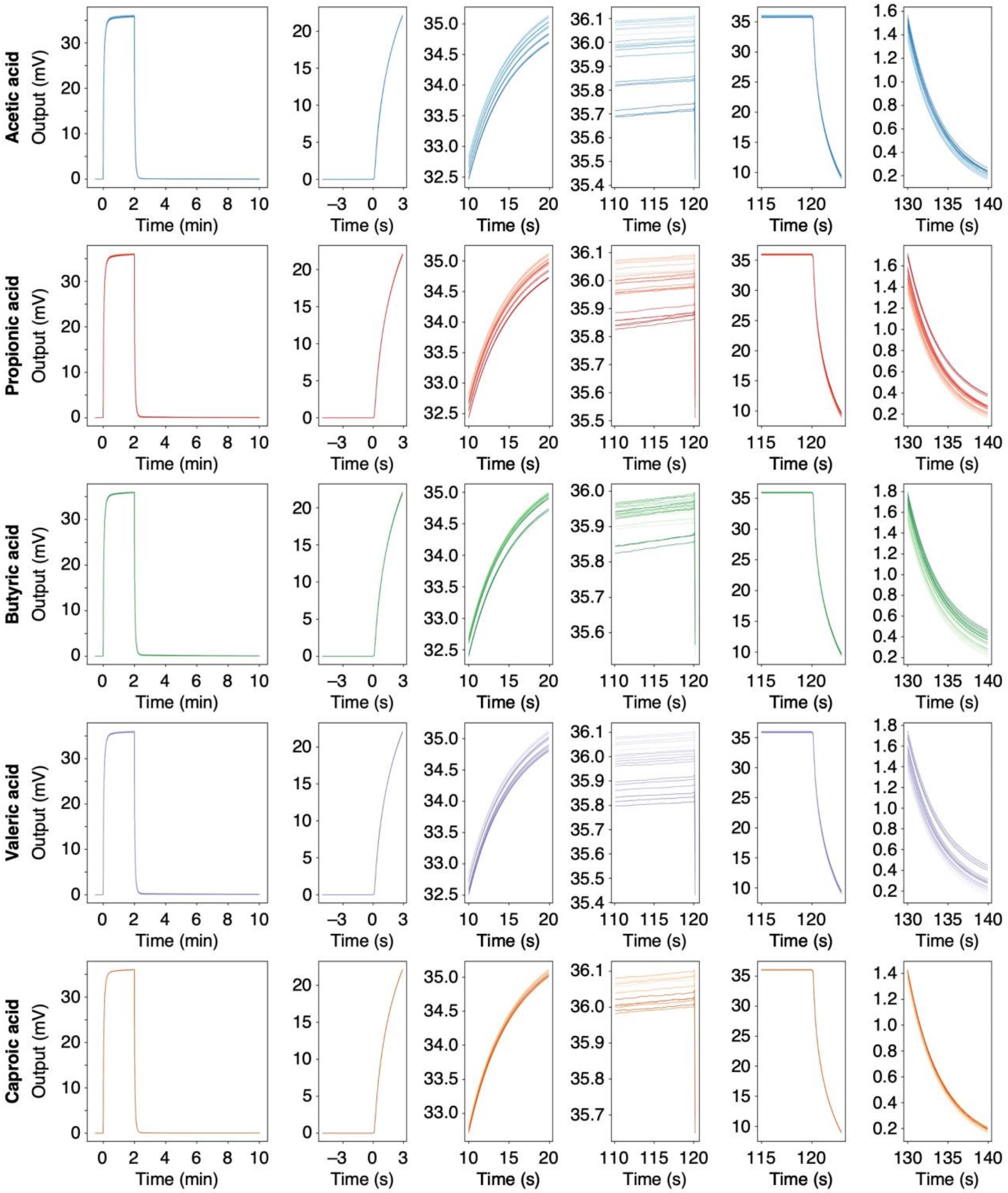

Figure S3. Signal responses of PMMA to various VFAs with different concentrations in water.

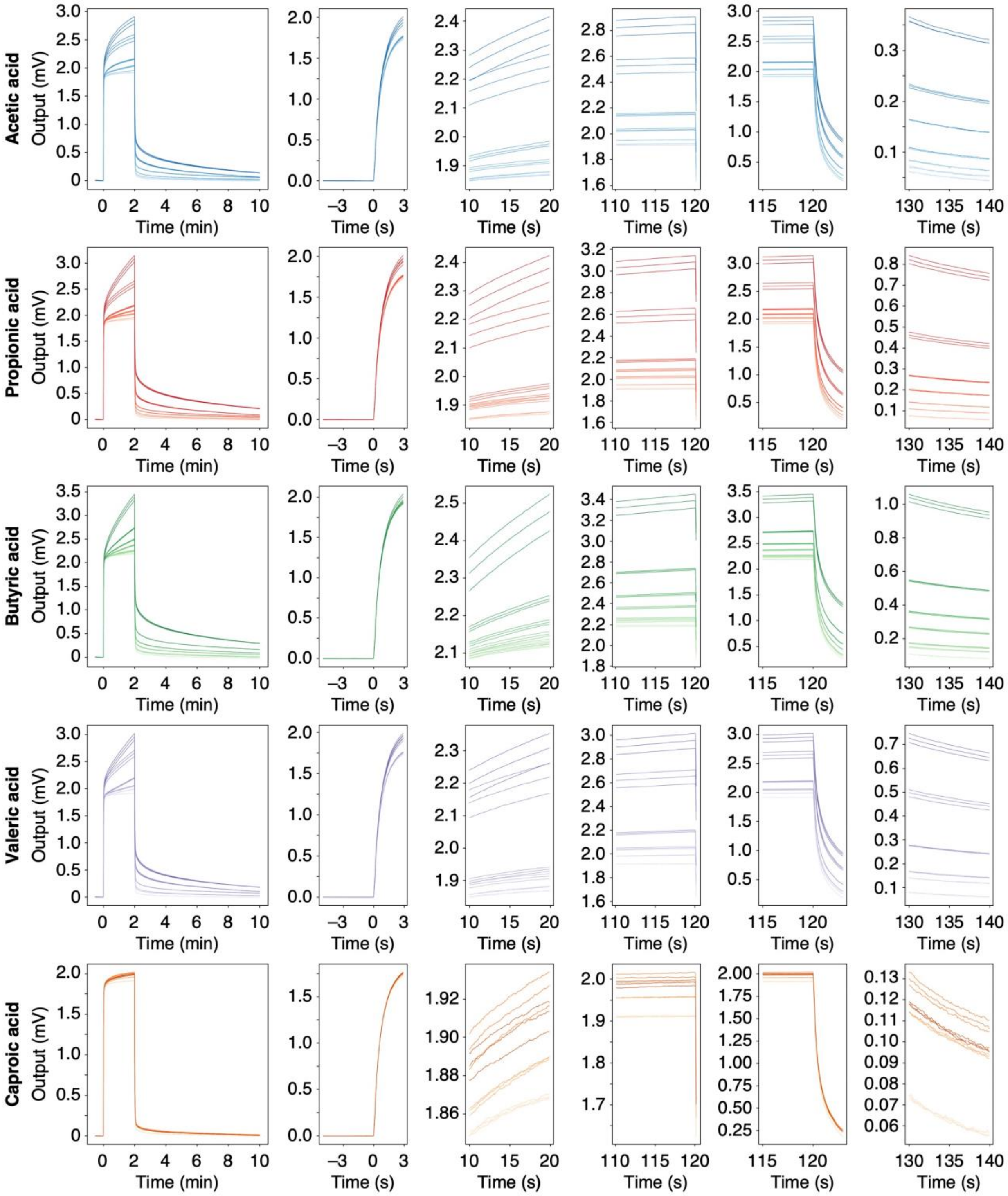

Figure S4. Signal responses of Tenax to various VFAs with different concentrations in water.

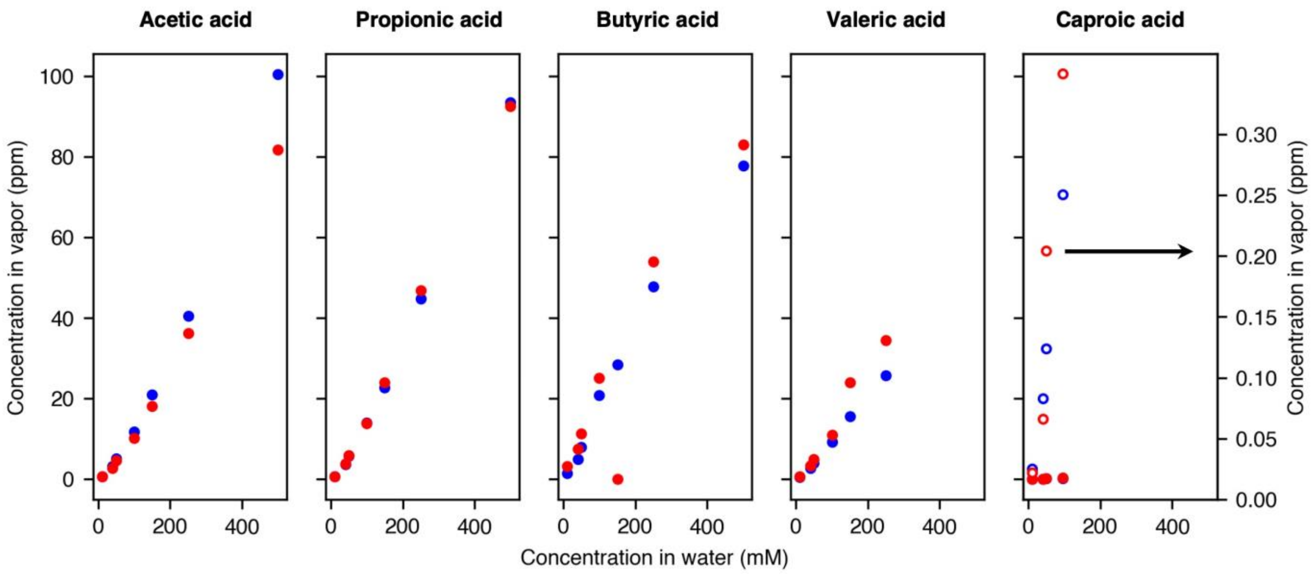

Figure S5. Vapor concentration of VFAs measured by PTR-TOF-MS. Blue and red circles denote the vapor concentration at 25 and 30 °C, respectively.

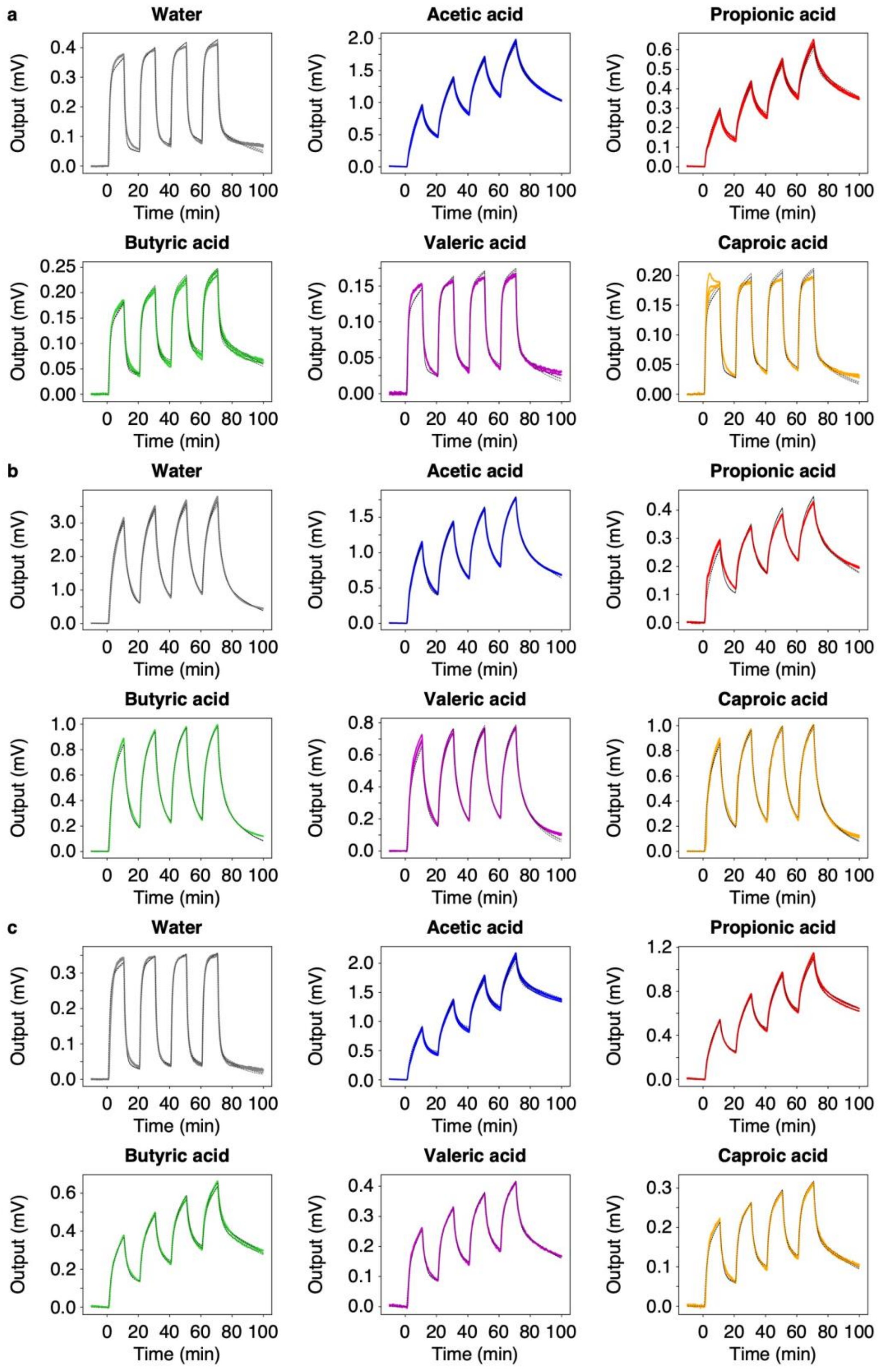

Figure S6. Estimation of diffusion time constants. Signal responses of PS (**a**), PMMA (**b**), and Tenax (**c**) to pure vapors of various VFAs without water vapors. Signal responses to water vapor are also presented. Solid lines and dashed lines represent measured signal responses and corresponding curve fitting results, respectively. Vapor concentrations are diluted by pure nitrogen and fixed at P / P^o = 10%, where P and P^o denote partial vapor pressure and saturated vapor pressure, respectively.

Supplementary Tables

Table S1. Results of chemical analysis of silage samples.

Silage	Plant	Water (%)	pH	LA [a] (%)	SA [a] (%)	MA [a] (%)	VFAs C_2 (%)	C_3 (%)	C_4 (%)	C_5 (%)	VBN [a] (mg N/g) [b]	T-N [a] (mg N/g) [b]
S1-1	Corn (Upper) [c]	65.9	4.41	0.52	0.06	0.20	0.71	0.10	0.09	0.00	0.434	3.827
S1-2		66.8	4.25	0.98	0.09	0.21	0.73	0.15	0.10	0.00	0.445	3.491
S1-3		67.0	3.98	1.31	0.11	0.19	1.00	0.16	0.13	0.00	0.442	3.770
S2-1	Corn (Bottom) [c]	66.3	3.78	2.03	0.16	0.19	0.95	0.05	0.08	0.00	0.473	3.934
S2-2		67.4	3.76	2.26	0.17	0.23	1.04	0.10	0.08	0.00	0.524	3.417
S2-3		65.9	3.75	2.20	0.15	0.20	1.06	0.09	0.08	0.00	0.497	4.026

[a] Abbreviations: LA, lactic acid; SA, succinic acid; MA, malic acid; VBN, volatile basic nitrogen; T-N, total nitrogen.
[b] Unit mg N/g indicates mg nitrogen per gram.
[c] Sampling position of silo

Table S2. Summary of signal outputs of PS to silage samples at $t = t_i$ (i = 1–5).

Sample	$t = t_1$ (mV) (%) [a]	$t = t_2$ (mV) (%) [a]	$t = t_3$ (mV) (%) [a]	$t = t_4$ (mV) (%) [a]	$t = t_5$ (mV) (%) [a]
Water	1.4011 ± 0.0038<br>0.24%	1.5538 ± 0.0011<br>0.07%	1.5864 ± 0.0002<br>0.01%	0.2958 ± 0.0040<br>0.25%	0.0676 ± 0.0015<br>0.10%
S1-1	1.4075 ± 0.0038<br>0.23%	1.5637 ± 0.0022<br>0.13%	1.6804 ± 0.0035<br>0.21%	0.3908 ± 0.0009<br>0.05%	0.1496 ± 0.0011<br>0.07%
S1-2	1.4022 ± 0.0046<br>0.26%	1.5757 ± 0.0024<br>0.14%	1.7641 ± 0.0023<br>0.13%	0.4758 ± 0.0023<br>0.13%	0.2242 ± 0.0005<br>0.03%
S1-3	1.4001 ± 0.0036<br>0.20%	1.5825 ± 0.0013<br>0.07%	1.7804 ± 0.0018<br>0.10%	0.4939 ± 0.0022<br>0.12%	0.2407 ± 0.0008<br>0.05%
S2-1	1.4196 ± 0.0048<br>0.24%	1.6297 ± 0.0035<br>0.17%	2.0152 ± 0.0118<br>0.59%	0.6955 ± 0.0081<br>0.40%	0.4056 ± 0.0069<br>0.34%
S2-2	1.4272 ± 0.0052<br>0.25%	1.6598 ± 0.0035<br>0.17%	2.0553 ± 0.0092<br>0.45%	0.7327 ± 0.0048<br>0.23%	0.4380 ± 0.0053<br>0.26%
S2-3	1.4228 ± 0.0049<br>0.24%	1.6553 ± 0.0034<br>0.17%	2.0472 ± 0.0105<br>0.51%	0.7291 ± 0.0069<br>0.34%	0.4351 ± 0.0069<br>0.34%

[a] Relative standard deviation defined by standard deviation / mean signal intensity ($t = t_3$)

Table S3. Summary of signal outputs of PMMA to silage samples at $t = t_i$ (i = 1–5).

Sample	$t = t_1$ (mV) (%) [a]	$t = t_2$ (mV) (%) [a]	$t = t_3$ (mV) (%) [a]	$t = t_4$ (mV) (%) [a]	$t = t_5$ (mV) (%) [a]
Water	18.1012 ± 0.0513<br>0.14%	32.1754 ± 0.0368<br>0.10%	35.4044 ± 0.0211<br>0.06%	12.8292 ± 0.0419<br>0.12%	1.5923 ± 0.0129<br>0.04%
S1-1	18.1600 ± 0.0630<br>0.18%	32.1927 ± 0.0446<br>0.13%	35.3517 ± 0.0282<br>0.08%	12.9143 ± 0.0327<br>0.09%	1.6711 ± 0.0211<br>0.06%
S1-2	18.0344 ± 0.0684<br>0.19%	32.0631 ± 0.0469<br>0.13%	35.3317 ± 0.0254<br>0.07%	13.0280 ± 0.0417<br>0.12%	1.7568 ± 0.0150<br>0.04%
S1-3	17.9631 ± 0.0671<br>0.19%	31.9837 ± 0.0538<br>0.15%	35.3171 ± 0.0334<br>0.09%	13.0516 ± 0.0289<br>0.08%	1.7763 ± 0.0177<br>0.05%
S2-1	17.9872 ± 0.0613<br>0.17%	32.0182 ± 0.0421<br>0.12%	35.4193 ± 0.0240<br>0.07%	13.1878 ± 0.0303<br>0.09%	1.8808 ± 0.0163<br>0.05%
S2-2	17.9305 ± 0.0544<br>0.15%	31.9558 ± 0.0442<br>0.12%	35.4325 ± 0.0252<br>0.07%	13.2194 ± 0.0401<br>0.11%	1.9155 ± 0.0183<br>0.05%
S2-3	17.9124 ± 0.0594<br>0.17%	31.9234 ± 0.0489<br>0.14%	35.3932 ± 0.0342<br>0.10%	13.2378 ± 0.0323<br>0.09%	1.9206 ± 0.0168<br>0.05%

[a] Relative standard deviation defined by standard deviation / mean signal intensity ($t = t_3$)

Table S4. Summary of signal outputs of Tenax to silage samples at $t = t_i$ (i = 1–5).

**Sample**	$t = t_1$ (mV) (%) [a]	$t = t_2$ (mV) (%) [a]	$t = t_3$ (mV) (%) [a]	$t = t_4$ (mV) (%) [a]	$t = t_5$ (mV) (%) [a]
Water	1.6483 ± 0.0037<br>0.19%	1.9135 ± 0.0021<br>0.11%	1.9818 ± 0.0004<br>0.02%	0.3514 ± 0.0043<br>0.22%	0.0803 ± 0.0016<br>0.08%
S1-1	1.6539 ± 0.0042<br>0.20%	1.9243 ± 0.0025<br>0.12%	2.1219 ± 0.0039<br>0.18%	0.4866 ± 0.001<br>0.05%	0.1963 ± 0.0029<br>0.14%
S1-2	1.6505 ± 0.0051<br>0.23%	1.9412 ± 0.0014<br>0.06%	2.2316 ± 0.0031<br>0.14%	0.592 ± 0.001<br>0.04%	0.288 ± 0.0012<br>0.05%
S1-3	1.6519 ± 0.0045<br>0.20%	1.9565 ± 0.0023<br>0.10%	2.2615 ± 0.0025<br>0.11%	0.6192 ± 0.0017<br>0.07%	0.3125 ± 0.0001<br>0.00%
S2-1	1.6784 ± 0.0055<br>0.20%	2.023 ± 0.0057<br>0.21%	2.668 ± 0.0197<br>0.74%	0.9645 ± 0.0143<br>0.53%	0.6111 ± 0.0131<br>0.49%
S2-2	1.6957 ± 0.0056<br>0.20%	2.0705 ± 0.0062<br>0.23%	2.7426 ± 0.017<br>0.62%	1.0216 ± 0.0107<br>0.39%	0.6611 ± 0.0114<br>0.42%
S2-3	1.6964 ± 0.0057<br>0.21%	2.0703 ± 0.0063<br>0.23%	2.7293 ± 0.0186<br>0.68%	1.0113 ± 0.0122<br>0.45%	0.6479 ± 0.0120<br>0.44%

[a] Relative standard deviation defined by standard deviation / mean signal intensity ($t = t_3$)
